# Supplementary material for: Oropouche Virus Infects, Persists and Induces IFN Response in Human Peripheral Blood Mononuclear Cells as Identified by RNA PrimeFlow™ and qRT-PCR Assays
Source: Viruses. 2020 Jul 21;12(7):785. doi: 10.3390/v12070785 (PMC7411765; doi:10.3390/v12070785)
Supplement: Supplementary file 1 [file viruses-12-00785-s001.pdf]

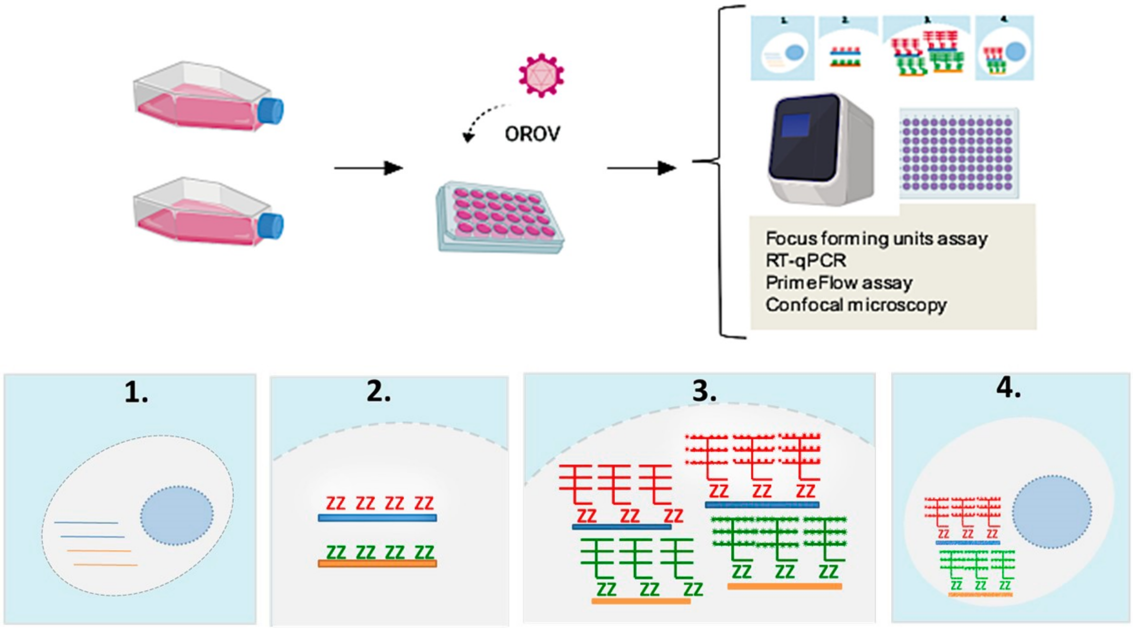

Supplemental Figure 1 (S1). Illustration of the workflow. Vero cells were seeded in 24 well plates and infected with OROV at MOIs 1 and 10. The cells were collected, and infection was evaluated by Focus forming units assay, RT-qPCR and confocal microscopy to compare with RNA PrimeFlow™ assay evaluation. A brief RNA PrimeFlow™ assay workflow is illustrated. 1. fixation and permeabilization of the cells; 2. RNA hybridization; 3. Three steps of signal amplification; 4. Acquisition.

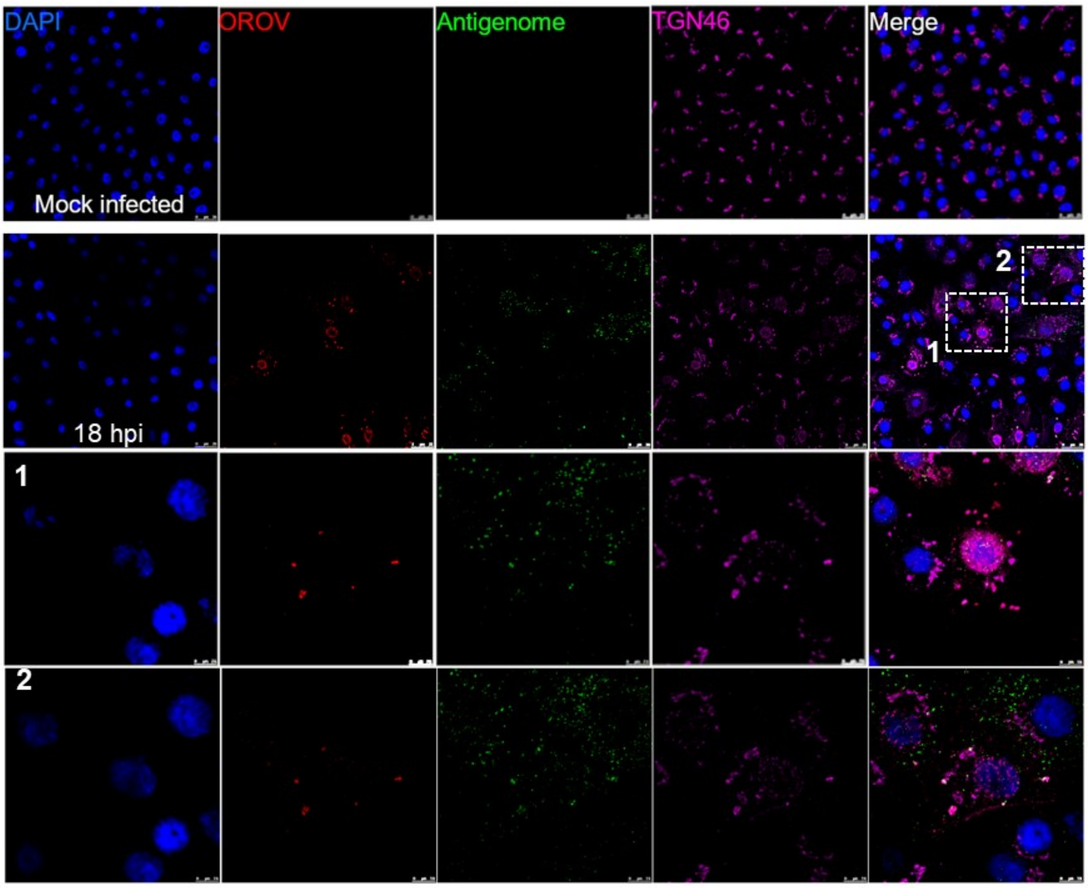

Supplemental Figure 2 (S2). OROV anti-genome colocalize with Golgi TNG46 proteins. Vero cells were cultivated in coverslips, infected at MOI 2 and submitted to RNA PrimeFlow™ protocol for immunofluorescence 18 hours post-infection. OROV proteins were stained with primary mouse fluid ascitic polyclonal anti-OROV antibodies and anti-mouse IgG conjugated with Alexa Fluor 647 antibodies (red); Antigenome probes are conjugated to Alexa Fluor 488 (green); TGN46 proteins were stained with sheep anti-TGN46 primary monoclonal antibodies and anti-sheep IgG conjugated with Alexa Fluor 594 (represented in gray), showing the viral factories. Nuclei were stained with DAPI (blue). Scales at 2 μm, with 10 μm in zoomed images 1 and 2.

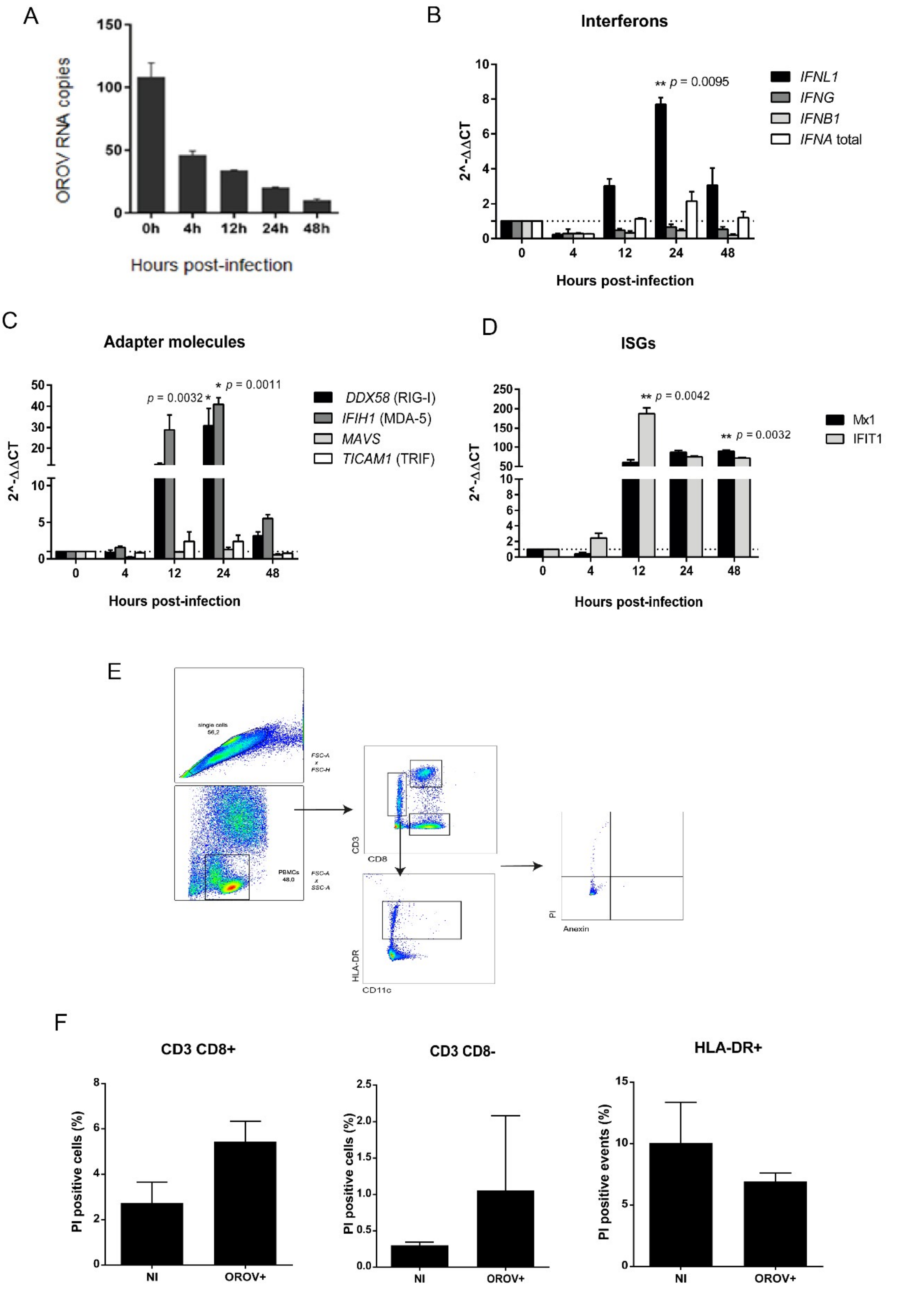

Supplemental Figure 3 (S3). *Ex vivo* infection of human whole blood with OROV. Whole blood were obtained from healthy donors diluted with RPMI medium 1:1 and infected *ex vivo* at 0, 2, 4, 12, 24 and 48 hours post-infection for RNA extraction and RT-qPCR. (A) OROV RNA copies detected by RT-qPCR. (B, C and D) SYBR Green qRT-PCR data analysis of gene expression are shown in fold change (2^ΔΔCT) and compared to the time 0 hours (non-infected). (E) Gating strategy for cell viability assay and (F) Percentages of PI positive cells. No Annexin event was detected. All infection kinetics experiments were performed in triplicates. Graphics show the median and standard error (SEM). All times post-infection were compared to the non-infected samples, or time 0, by One-way ANOVA and Dunn's multiple comparisons test.

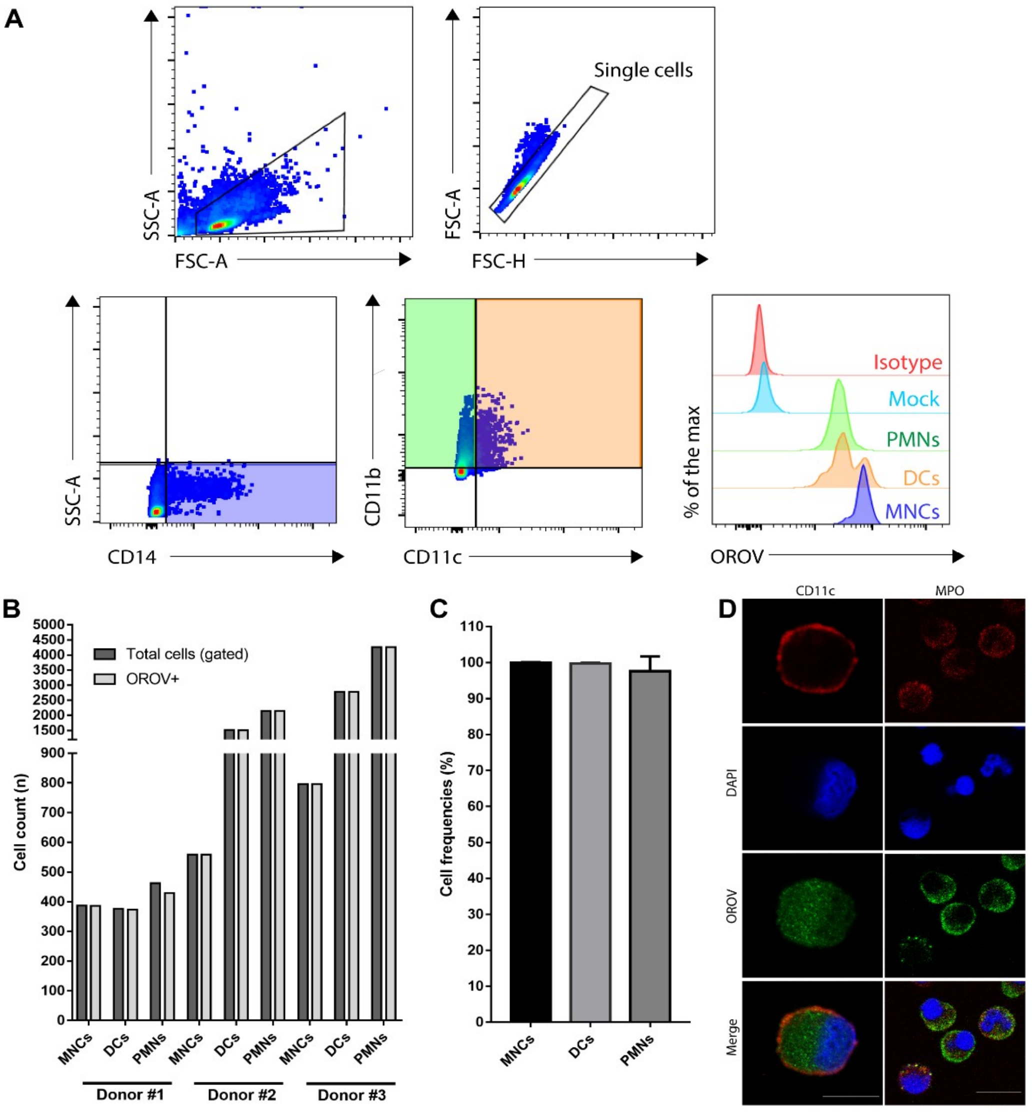

Supplemental Figure 4 (S4). Detection of OROV antigens in myeloid cells infected *in vitro*. (A) Gating strategies used to analyze myeloid cells susceptibility to OROV infection. PBMCs from healthy donors (n = 3) were purified by density gradient and infected in vitro with OROV in a MOI of 1. After 24h, cells were first labelled using CD14, CD11c and then fixed, permeabilized and stained with polyclonal anti-OROV. (B) Cell count and (C) frequency of MNCs, DCs and PMNs presenting OROV antigens. Bars represent cell count and frequency ± SEM. (D) Phenotype of OROV antigen distribution in the main target cells. At 24hpi, PBMCs were plated into coverslips and stained with anti-CD11c or anti-MPO (red), anti-OROV (green) and nuclei (blue). Coverslips were analyzed in confocal microscopy. Scale bar: 20 μm.
